# DJ-1 Knockout Augments Disease Severity and Shortens Survival in a Mouse Model of ALS

**DOI:** 10.1371/journal.pone.0117190

**Published:** 2015-03-30

**Authors:** Nirit Lev, Yael Barhum, Itay Lotan, Israel Steiner, Daniel Offen

**Affiliations:** 1 Neuroscience Laboratory, Felsenstein Medical Research Center, Tel Aviv University, Petah Tikva, Israel; 2 Department of Neurology, Rabin Medical Center, Tel Aviv University, Petah Tikva, Israel; University of Florida, UNITED STATES

## Abstract

Amyotrophic lateral sclerosis (ALS) is a progressive, lethal, neurodegenerative disorder, characterized by the degeneration of motor neurons. Oxidative stress plays a central role in the disease progression, in concert with an enhanced glutamate excitotoxicity and neuroinflammation. DJ-1 mutations, leading to the loss of functional protein, cause familial Parkinson’s disease and motor neuron disease in several patients. DJ-1 responds to oxidative stress and plays an important role in the cellular defense mechanisms. We aimed to investigate whether loss of functional DJ-1 alters the disease course and severity in an ALS mouse model. To this end we used mice that express the human SOD1^G93A^ mutation, the commonly used model of ALS and knockout of DJ-1 mice to generate SOD1 DJ-1 KO mice. We found that knocking out DJ-1in the ALS model led to an accelerated disease course and shortened survival time. DJ-1 deficiency was found to increase neuronal loss in the spinal cord associated with increased gliosis in the spinal cord and reduced antioxidant response that was regulated by the Nrf2 mechanism.The importance of DJ-1 in ALS was also illustrated in a motor neuron cell line that was exposed to glutamate toxicity and oxidative stress. Addition of the DJ-1 derived peptide, ND-13, enhanced the resistance to glutamate and SIN-1 induced toxicity. Thus, our results maintain that DJ-1 plays a role in the disease process and promotes the necessity of further investigation of DJ-1 as a therapeutic target for ALS.

## Introduction

Amyotrophic lateral sclerosis (ALS) is a progressive, lethal, neurodegenerative disorder, characterized by the degeneration of motor neurons in the brain and spinal cord. Death of the upper motor neurons leads to spasticity, hyperexcitability of reflexes and the appearance of pathological reflexes, such as Babinski reflex. The death of the lower motor neurons leads to weakness and atrophy of the muscles followed by progressive paralysis [1, 2]. Respiratory failure is the common cause of death, which typically occurs within 2–5 years from disease onset. The disease occurs worldwide with an annual incidence of 1–2.5 per 100,000, primarily affecting adults with onset at a mean age of 60–65 [3]. Most ALS patients suffer from a sporadic disease. However, in the recent years, it has been recognized that about ten percent of ALS cases have a familial cause (fALS); of which about 20% are caused by a mutation in the gene-encoding Cu/Zn superoxide dismutase (SOD1) on chromosome 21q22 [4].This genetic information helped in the developmentof animal models for the study of ALS. The most commonly used rodent model of ALS is the transgenic rodent harboring the G93A mutation, in which the amino acid glycine is replaced by alanine at position 93 of human SOD1 (SOD1 mice). These mice imitate much of the human ALS phenotype as they lose motor neurons, develop progressive paralysis anddisplay a shortened survivalrate [5].

Although the causes of ALS are still unknown, several hypotheses have been postulated, including oxidative-nitrosativestress, mitochondrial dysfunction, excitotoxicity,neuroinflammation, protein misfolding, neurotrophic factors deficiency, and altered axonal transport. In humans, as well as animal models, it has been shown that oxidative stress plays a central role in the progression of motor neuron loss, possibly in concert with a chronically enhanced excitotoxicity and neuroinflammation [6,7]. Excitotoxicity mediated by glutamate has been implicated in the selective susceptibility of motor neurons occurring in ALS. Overstimulation of glutamate receptors on motor neurons cause cell death through an increase of cytosolic free calcium, mitochondrial damage and activation of death cascades [8]. Glutamate excitotoxicity and reactive oxygen species (ROS) are interconnected. ROS can reduce the uptake of glutamate in mammals; however, increased calcium levels in the mitochondria due to dysfunctional glutamate regulation can result in overproduction of ROS and cause oxidative stress [9]. The question remains whether oxidative stress causes glutamate dysregulation or vice versa.

DJ-1 encodes a small 189 amino acid protein that is ubiquitously expressed and highly conserved throughout diverse species [10,11]. DJ-1 is widely distributed and is highly expressed in the central nervous system (CNS) and is not confined to a single anatomical or functional system [12]. DJ-1 mutations are known to cause early onset autosomal recessive Parkinson’s disease (PD) [13]. Several mutations are currently known, all of which lead to loss of functional protein. A Sicilian family was found in which three brothers with DJ-1 mutations suffered from symptoms of both PD and motor neuron disease [14,15]. Accumulating evidence suggests that DJ-1 responds to oxidative stress [16,17]. Upon exposure to oxidative stress, oxidation of cysteine and methionine residues in DJ-1 protein cause a shift of DJ-1 isoelectric point (pI) and appearance of more acidic isoforms [18,19]. This shift in DJ-1's pIwas notedin human brains in postmortem samplestaken from PD patients and compared to controls [20].

We havepreviously shown that in an animal model of ALS, SOD1 transgenic mice, there are significant changes in DJ-1 expression and in its acidic isoforms [21]. Furthermore,there was a correlation between DJ-1 levels andclinical disease progression [21]. Alterations in DJ-1 were also found in the cerebrospinal fluid (CSF) of ALS patients [22]. Moreover, Wang et alfound that DJ-1 affects normal SOD1 expression [23]. Collectively, these findings support the need of functionalDJ-1 for appropriate protection against neurotoxicinsultsin ALS.

The aim of the current study was to further examine the role of DJ-1 in ALS.We created double transgenic mice: DJ-1 knockout mice that also express the human SOD1 mutation. We thereby examined whether loss of DJ-1 affectsthe disease course and survival*in vivo*.Possible abilities of DJ-1 were tested *in vitro* utilizing a DJ-1 derived peptide (ND-13) and found to be protective in stress conditions.

## Materials and Methods

### Transgenic mice

This study was carried out in strict accordance with the recommendations in the Guide for the Care and Use of Laboratory Animals of the National Institutes of Health. The protocol was approved by the Committee on the Ethics of Animal Experiments of the University of Tel Aviv (Permit Number: M-10045). Mice overexpressing the human mutant G93A SOD1 (SOD1) and DJ-1 knockout (DJ-1 KO) were purchased from Jackson Laboratories (Bar Harbor, Maine, USA). The SOD1 transgenic mouse model was developed and characterized as a model for ALS by Gurney et al. 1994 [24]. The animals were housed in standard conditions: constant temperature (22±1°C), humidity (relative, 40%), and a 12-h light/dark cycle and were allowed free access to food and water. Male mice with hemizygous mutated SOD1 were bred with C57/bl6 female. At one month of age, offspring were genotyped by PCR analysis to confirm their transgenic status.

To generate double transgenic mice, SOD1 males were bred with DJ-1 KO females. Males that were identified as heterozygous for DJ-1 knockout + SOD1 were then bred with DJ-1 KO females. The offspring of these mice that were identified as DJ-1 knockout and contained mutant SOD1 were chosen for further studies (termed herein as SOD1 DJ-1 KO), and compared to SOD1 mice with normal DJ-1.We used wild type (WT) littermates and DJ-1 KO mice as control groups. Both males and females were included in the experiments. However, since prior experience has shown that the gender affects disease course,we analyzed males and females separately.

Evaluation of disease progression and severity included both clinical, biochemical and histological analysis. Disease course, survival and behavioral analysis were performed on 54 mice, SOD1 (9 female, 9 male), and SOD1 DJ-1 KO mice (14 female and 22 male). Biochemical and histological analysis was done at 3 stages of the disease progression: pre-clinical (10 weeks, day 70), clinical (15 weeks, day 105) and end-stage disease, n = 5 per group at each time point for each analysis. Age-matched WT mice and DJ-1 knockout mice were included in the biochemical and histological analysis.

### Clinical evaluation

Mice body weight was measured on a weekly basis, starting at the age of 5 weeks, and twice weekly when early disease signs appeared. Disease onset was calculated retrospectively as the day the mouse reached peak body weight. The mice were followed-up closely and age of death was registered in order to examine if DJ-1 knockout affects survival. To determine mortality in a reliable and humane fashion, we used an artificial end point, defined as the inability of the mice to right themselves 30 seconds after being placed on one of their sides, as done in previous studies.

### Behavioral examination

Starting at the age of 5 weeks, mice were examined weekly for signs of motor impairment. Two clinical disease scales were used in order to assess clinical disease progression. One scale was used to score progressive gait difficulties and another score was used to evaluate tail-hanging hindlimb splay reflex. Starting at day 100 these behavioral scores were evaluated twice weekly. Gait was evaluated using a well accepted 5-point behavioral score system [25].

Gait was scored as follows:healthy without any symptoms of paralysis (score—5); slight signs of destabilized gait and paralysis of the hind limbs (score—4); obvious paralysis and destabilized gait (score—3); fully developed paralysis of the hind limbs, animals only crawling on their forelimbs (score—2); fully developed paralysis of the hind limbs, animals predominantly lying on their side and/or were not able to straighten up after turning them on the back or lost more than 20% of their initial weight (score—1). When animals reached a score of 2 macerated food was given daily for easy access of food and hydration.

Mice were gently lifted from their tails and the hindlimb splay reflex was observed for tremors, rigidity and the ability to extend both limbs. The reflex was scored with a clinical scale of 5 (normal) to 1 (severely pathologic). In the Rotarod test, following a brief training period, adult wild-type mice were able to remain balanced on a rotating rod atan accelerated speed, from 0 to 25 RPM/min, for up to 4 minutes. Each session consistedof three trials on the rotarod, and the time each mouse remained on the rotarod was registered.

### Biochemical evaluation

The mice were sacrificed, and their spinal cords were removed, rapidly frozen in fluid nitrogen and kept in -80°C for RNA and protein isolation and further analysis. Total RNA was isolated from mice brain tissues using a commercial reagent TriReagent (Sigma-Aldrich) and the manufacturer’s recommended procedure. The amount of RNA was determined spectrophotometrically using the ND-1000 spectrophotometer (Nano-drop, Wilmington, DE, UASA). RNA quality was verified by measuring OD260/OD280 ratio. RNA was stored at −80°C until used. First-strand cDNA synthesis was carried out as described before (18, 21). cDNA was prepared to a final reaction volume of 20 μl containing 1 μg of the total RNA, random primer (1.3 μM, Invitrogen, UK) in DEPC-treated water at a total volume of 10 μl. After incubation in 70°C for 10 minutes and cooling to 4°C for 10 minutes, the following reagents were added to a final concentration: 1× buffer supplied by the manufacturer,10 mM DTT, 20 μM dNTPs, 20 U of RNase inhibitor (RNAguard, Amersham Pharmacia Biotech, Piscataway, NJ), and 10 U of the enzyme Super Script III RNase H-reverse transcriptase (Invitrogen). RT reaction was performed at 25°C for 10 minutes, 42°C for 2 hours followed by 70°C for 15 minutes and 95°C for 15 minutes. Samples were stored at −20°C until used.

Real-time quantitative reverse transcription polymerase chain reaction (PCR) of the desired genes was performed in an ABI Prism 7700 sequence detection system (Applied Biosystems) using Sybr green PCR master mix (Applied Biosystems). Analysis of mRNA expression levels of genes of interest was quantified compared to the housekeeping genes, GAPDH utilizing the ΔΔCT method. Primers used include:

GLT-1: CAG TGC TGG AAC TTT GCC TG Forward,
GLT-1: GGC TAT GAA GAT GGC TGC CA Reverse,
GAPDH: CCA TGG AGA AGG CTG GGG Forward,
GAPDH: CAA AGT TGT CAT GGA CC Reverse.

Proteins were extracted from brain tissues as described before (21), by grinding 10 mM KCl,1.5 mM MgCl_2_, 2 mM ethylene diaminetetraacetic acid, 20 mM (4-(2-hydroxyethyl)-1-piperazineethanesulfonic acid and protease inhibitors cocktail (Roche) in lysis buffer containing 250 mM sucrose. Cell debris were removed by centrifugation at 20,000×g for 15 minutes at 4°C. Protein concentration was determined by the BCA method (Pierce,Rockford, IL, USA). Twenty-five micrograms of total protein frombrain sample lysates was separated by 12% sodium dodecyl sulfate polyacrylamide gel electrophoresesand transferred to nitrocellulose membranes. The membranes were probed with antibodies anti-ChAT (Santa Cruz Biotechnology Inc. Dallas TX, USA), anti-Nrf2 (Santa Cruz), anti-hemeoxygenase 1 (HO-1, Enzo, Town, Country), anti-GFAP (Dako), and anti beta-actin (Sigma) antibodies. Analysis was done using the Odyssey CLx Western Blot Detection System,the Netherlands.

### Histology

Mice were sacrificed, at different stages of the disease, and their spinal cords removed and immersed in 4% paraformaldehyde (pH 7.4) overnight. For Nissl staining, the lumbar spinal cords were embedded in paraffin and then deparaffinized using xylene and a graded alcohol series. Lumbar spinal cord sections were obtained at 30 μm intervals and stained with Nissl staining in order to evaluate motor neurons. To compare the number of residual motor neurons, we counted the number of large neurons (greater than 25 μm in diameter) in the ventral horn of the lumbar spinal cord in three Nissl-stained sections.

### Cell cultures

Mouse NSC-34 hybrid cell line, a widely used motor neuron-neuroblastoma fusion line was chosen, as it expressed many of the morphological and physiological properties of motor neurons [26]. Cellswere grown in Dulbecco’s modified Eagle’s (DMEM)/F-12 medium supplemented with 10% fetal calf serum and 1% of a mixture of penicillin/streptomycin at 37°C in an atmosphere of 5% CO2 in air. Human neuroblastoma cells, SH-SY5Y cells were obtained from the ATCC (Rockville, USA), and were grown on tissue culture plates (Greiner, Hessle, UK) in DMEM, supplemented with 10% fetal calf serum, 1% L-glutamine and 1% SPN antibiotics (Biological Industries, Bet Haemek, Israel). Cells were incubated at 37°C in a humidified atmosphere with 5% CO_2_. Cells were exposed to peroxynitrite ion generator 3-morpholinosydnonimine (SIN-1, Sigma–Aldrich) or to glutamate (Sigma—Aldrich).

### ND-13 a DJ-1 based peptide

We made athorough bio-informatic survey and used the knowledge gained in previous studies to locate conserved areas in the DJ-1 protein. We then designed a series of short peptides derived from DJ-1 and using an *in vitro* cellular platform based on human neuroblastoma SH-SY5Y cells,we chose a peptide that effectively protected against oxidative and toxic insults. The most promising peptide, named ND13, is composed by 13 amino-acids [KGAEEMETVIPVD]. In order to enable cell penetration, we attached a 7 amino-acids sequence of cell penetrating peptide (CPP) derived from the HIV TAT protein [YGRKKRR] [27]. As a control peptide we used scrambled amino acids sequence peptide (13 amino acids in the opposite direction) attached to the same CPP moiety.

### Alamar blue assay

The protective effect of a DJ-1-based peptide was evaluated with and without exposure to SIN-I by the Alamar-blue method as we described previously [18]. Briefly, cells were seeded in 96-wells plates at a concentration of 5000 cells per well and allowed to attach overnight. On the following day, the cells were exposed to increasing doses of SIN-I (0–0.5mM, Sigma–Aldrich) for 24 hours, with or without pretreatment with DJ-1-based peptide, ND-13 (10 µM, applied one hour before toxic exposure). All experiments were done in serum free medium. The reduction-induced color change of Alamar-blue varies proportionately with cell number and time. A solution of alamar blue 10% in serum free medium was added for 2 hours and fluorescence was measured by FLUOstar spectrofluorometer at the excitation wavelength of 544 nm and the emission wavelength of 590 nm. Each experiment was done in triplicate for each treatment. The experiments were repeated 3 times.

### Apoptosis assessment by Annexin V- propidium iodide (PI) flow cytometric analysis (FACS)

The effects of ND-13 on glutamate-induced apoptosis were analyzed by Annexin V-FITC and PI staining using FACS analysis. To detect total apoptosis (early and late apoptosis), cells were cultured at an initial density of 3×10^5^ cells/well in 6-well plates and treated with glutamate (17mM), ND-13 (10 µM) or vehicle. ND-13 was applied 1 hour before exposure to glutamate toxicity. The cells were collected, centrifuged, and resuspended in binding buffer, and incubated with 10 µl of Annexin V-FITC (Sigma–Aldrich) and 10 µl PI solution for 30 minutes at room temperature in the dark. After Annexin V-FITC and PI double-staining, the cells were analyzed by flow cytometric analysis (FACS). The various stages of apoptosis were differentiated as follows: early apoptosis (Annexin V+/PI−); late apoptosis/necrosis cells (Annexin V+/PI+); and viable cells (Annexin V−/PI−).

### Statistical analysis

**Fig 1:**
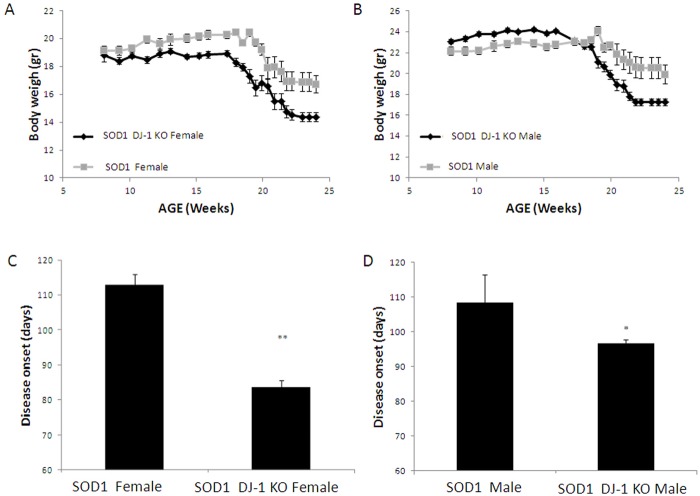
SOD1 DJ-1 KO-mice lost weight earlier and faster than SOD1 mice. A, Weight of female mice. B, Weight of male mice. Disease onset was calculated retrospectively as the day the mouse reached peak body weight. Disease onset was significantly earlier in both male and female SOD1 DJ-1 KO mice as compared to SOD1 mice (C, female mice; D, male mice). Data is presented as averages ± SE. * p<0.05, ** p<0.01. n = SOD1 mice 9F and 9M, SOD1 DJ-1 KO mice 14F and 22M.

**Fig 2:**
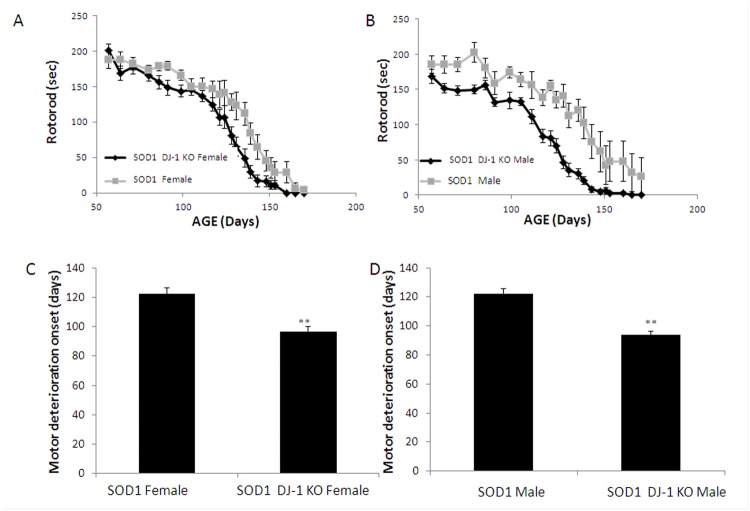
Motor function of SOD1 DJ-1 KO mice deteriorated faster than SOD1 mice. The motor function and deterioration was evaluated by Rotarod (A, female mice; B, male mice). The age at which the performance of the animals fell below 150 seconds was taken as an index ofdeteriorationonset. (C, females; D, males).Data is presented as averages ± SE. ** p<0.01. n = SOD1 mice 9F and 9M, SOD1 DJ-1 KO mice 14F and 22M.

**Fig 3:**
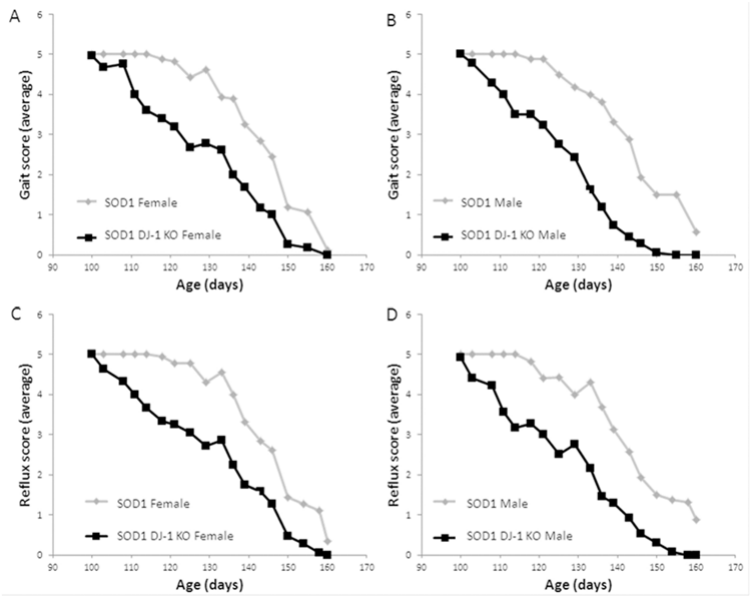
SOD1 DJ-1 KO mice demonstrated an accelerated disease course. Accelerated deterioration of gait was observed in SOD1 DJ-1 KO mice as compared to SOD1 mice. The score for gait of (A) female and (B) male mice was evaluated on a clinical scale of 5 (normal) to 1 (severely pathologic). Hind limb splay reflex of female (C) and male (D) mice p<0.05, SPSS repeated measures. n = SOD1 mice 9F and 9M, SOD1 DJ-1 KO mice 14F and 22M.

**Fig 4:**
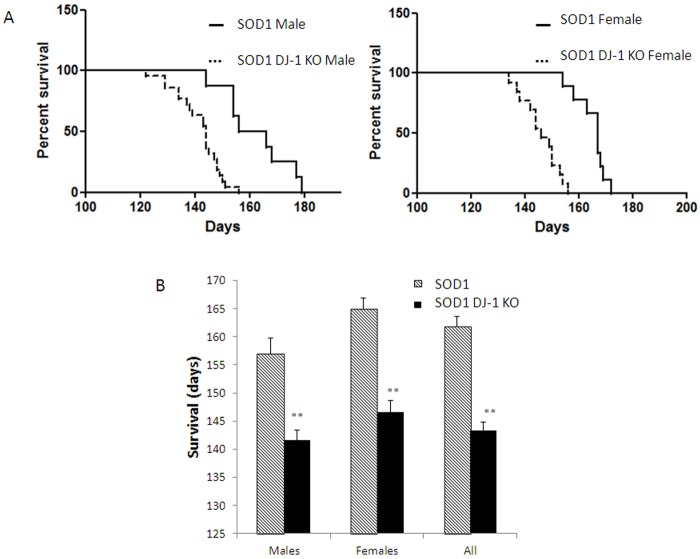
SOD1 DJ-1 KO mice demonstrated shortened survival. Kaplan-Meier survival curves of (A) male and female SOD1 DJ-1 KO mice and SOD1 mice.(B) The average survival (in days) of SOD1 DJ-1 KO mice and SOD1 mice is presented as averages ± SD. ** P<0.01. n = SOD1 mice 9F and 9M, SOD1 DJ-1 KO mice 14F and 22M.

**Fig 5:**
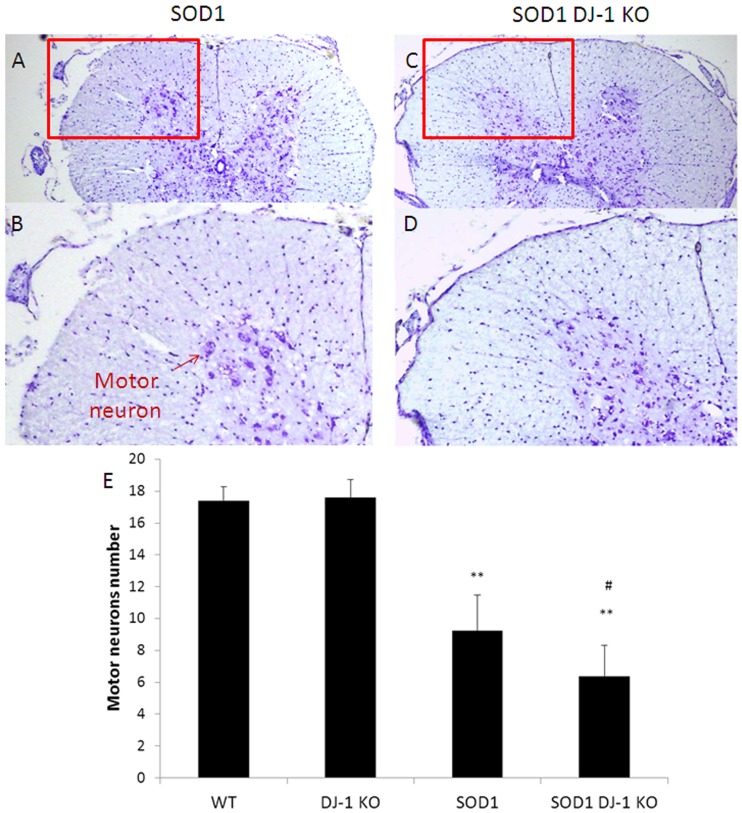
Ventral horn motor neuron survival. Lumbar spinal cord sections were stained by Nissl stain. Motor neurons were identified according to the criteria detailed in the methods section. At symptomatic disease stage (15 weeks), motor neurons loss was augmented in SOD1 DJ-1 KO as compared to SOD1 mice. Nissl Stain of lumbar spinal cord sections of SOD1 mice are presented in A (low magnification) and B (high magnification). Nissl Stain of lumbar spinal cord sections of SOD1 DJ-1 KO mice are presented in C (low magnification) and D (high magnification). The red circles signify the ventral horns, from which the high magnification pictures were taken. Quantification of motor neurons in the ventral horn of the lumbar spinal cord in the different groups is shown graphically (E). The values are presented as averages ± SD. ** p< 0.001 vs. WT mice; # p< 0.01 SOD1 DJ-1 KO mice vs. SOD1 mice.

**Fig 6:**
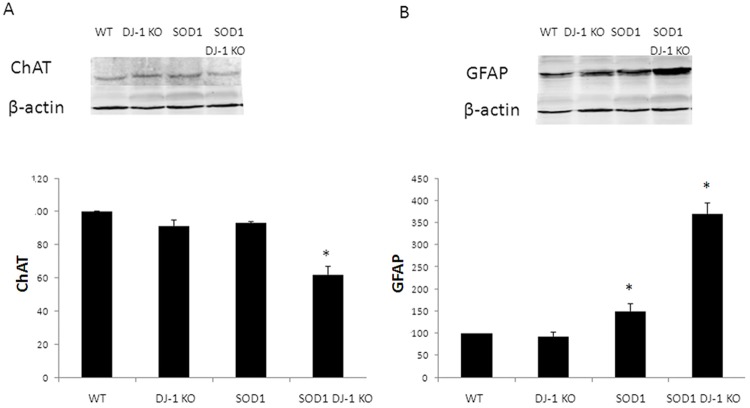
Motor neuron loss and astrogliosis in the spinal cords of SOD1 DJ-1 KO. Choline acyl transferase (ChAT, A) and glial fibrillary acidic protein (GFAP, B) levels were determined in the spinal cord extracts by Western blot analysis. Proteins levels were normalized to beta actin. Data is presented as averages ± SD. * p<0.05. n = 3 female and 2 males.

**Fig 7:**
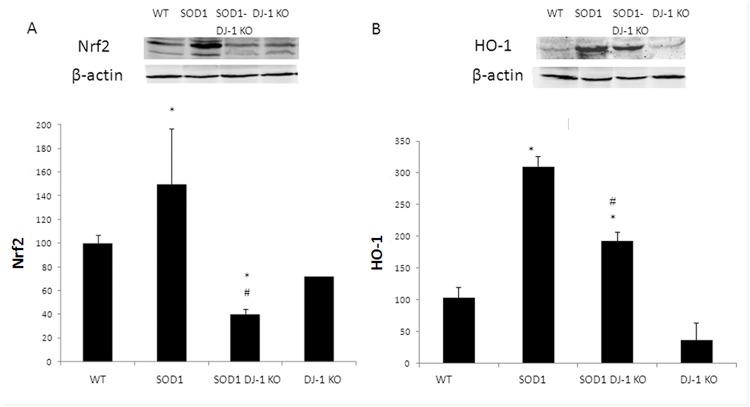
Attenuated Nrf2 system in SOD DJ-1 KO mice. Mutated SOD1 increased the expression of Nrf2 and HO-1 while SOD1 DJ-1 KO demonstrated reduction. Nuclear factor erythroid-2 related factor 2 (Nrf2, A) and hemeoxygenase 1(HO-1, B) protein levels were determined in the spinal cord extracts by Western blot anlysis. Proteins levels were normalized to beta actin. Data is presented as averages ± SD. * p<0.05. n = 3 female and 2 males.

Statistical analysis of data sets was carried out using SPSS for Windows (version 10.0.1). Statistical significance for Figs. 1–3 was determined by repeated measures. Statistical significance for Fig. 5–7 was determined by one-way ANOVA, followed by Scheffe's post hoc multiple comparisons. Error bars represent the standard error of the mean (SEM). The Kaplan-Mayer test was employed to determine the statistical significance for the survival test in Fig. 4. Significance was considered when p<0.05.

## Results

### Augmented disease severity in SOD1 DJ-1 KO mice

As disease progressed, mice started to lose weight, as has also been shown in previous studies. At the age of 20 weeks both mice groups showed a reduction in body weight. However, the SOD1 DJ-1 KO- mice lost weight significantly earlier and faster than the SOD1 mice, in both the male and female groups (Fig. 1A, B). As was done in previous studies, we defined disease onset at the age when mice reached their peak weight and started losing weight. Disease onset was significantly earlier in both male and female SOD1 DJ-1 KO mice as compared to SOD1 mice. In females, disease onset was at day 83 vs. 112 (p<0.01) while in males 96 vs. 108 days (p<0.05, Fig.1C, D) for SOD1 DJ-1 KO and SOD1, respectively. Several parameters were employed in order to quantify clinical disease progression and disease severity. Since the major system affected by the disease is the motor system, tests assessing motor performance were used. Ability to walk on an accelerating rotarod up to 4 minutes was used to assess both the onset of motor deterioration and the further progression of motor deficits (motor coordination, strength and balance). We also used clinical scoring systems in order to quantitate the progressive deterioration of gait and hindlimb splay reflex as disease progressed. As controls, we used WT and DJ-1 KO littermates. No significant differences were found between WT and DJ-1 KO mice in motor performance tests up to the age of 16 weeks.

Progressive motor deterioration was evident in both SOD1 and SOD1 DJ-1 KO mice groups. Recording runtime on the accelerating rotarod showed that motor function of both male and female SOD1 DJ-1 KO mice deteriorated faster than SOD1 mice (Fig. 2A, B). We determined the age at which the performance of the animals fell below 150 seconds for two consecutive measures, and this time point was taken as an index of rotarod impairment. Both male and female SOD1 DJ-1 KO mice demonstrated earlier motor deterioration as compared to SOD1 mice. The index point of rotarod impairment was reached at 97 days in female SOD1 DJ-1 KO mice vs. 122 days in female SOD1 mice (p<0.01) (Fig. 2C), and at the ages of 94 days vs. 122 days in males, respectively (p<0.01) (Fig. 2D). Clinical scoring of gait and hindlimb splay reflex parameters also showed accelerated deterioration in SOD1 DJ-1 KO mice as compared to SOD1 mice, with earlier and accelerated decline in these clinical scores in male and female SOD1 DJ-1 KO mice as compared to SOD1 mice (Fig. 3).

### SOD1 DJ-1 KO mice showed reduced survival

SOD1 DJ-1 KO mice demonstrated significantly reduced survival compared to SOD1 mice (Fig. 4). Kaplan-Meier survival curves of male and female SOD1 DJ-1 KO mice as compared to SOD1 mice are presented in Fig. 4A. Overall SOD1 DJ-1 KO mice had a mean survival of 143.4 days, which was significantly shorter than the survival of SOD1 littermates (a mean survival of 161.8 days, the difference of 18.4 days; p<0.01) (Fig. 4A). Female SOD1 DJ-1 KO mice had a mean survival of 146.6 days, as compared to 165 days in SOD1 females (a difference of 18.4 days; p<0.01) (Fig. 4B). Average survival of male SOD1 DJ-1 KO mice was 141.6 days compared to the average survival of male SOD1 mice of 157 days (a difference of 15.4 days; p<0.01) (Fig. 4B).

### Accelerated motor neurons loss and astrogliosisin the spinal cords of SOD1 DJ-1 KO

To evaluate the survival of motor neurons, lumbar spinal cord sections were assessed using the Nissl stain. No significant differences were observed in the number of motor neurons in the lumbar spinal cords of DJ-1 KO and WT mice. As was reported, at symptomatic disease stages motor neurons in the spinal cord were reduced in the SOD1 model. Motor neurons counting in the pre-symptomatic mice (day 70) did not reveal significant differences between SOD1 DJ-1 KO and SOD1 mice. At symptomatic disease stages (15 weeks), there was a significant loss of motor neurons in both SOD1 DJ-1 KO and SOD1 mice as compared to WT and to DJ-1 KO mice (53%±12.9 vs. 36.5%±11.31 of WT numbers, respectively. Fig. 5). SOD1 DJ-1 KO mice had augmented loss of motor neurons as compared to SOD1 mice (p<0.001. Fig. 5). The analysis of motor neurons numbers in animals at endstage disease revealed minor non significant differences between SOD1 DJ-1 KO and SOD1 mice.

To further evaluate the survival of motor neurons in the different mice groups we measured the choline acyl transferase (ChAT) expression in spinal cord extracts, using Western blot analysis. We demonstrated that the levels of ChAT in SOD1 DJ-1 KO decreased compared to those of SOD1 mice at the early symptomatic stage (day 90, Fig. 6A). In order to quantify astrogliosis in the lumbar spinal cord, Western blots were probed with anti-glial fibrillary acidic protein (GFAP) antibodies. During the symptomatic disease stage both SOD1 and SOD1 DJ-1 KO mice showed significantly increased astrogliosis in the lumbar spinal cord. However, a marked increase in astrogliosis was apparent in SOD1 DJ-1 KO, indicating an accelerated disease course (Fig. 6B).

### Activation of Nrf2 and alteration in the glutamate system in the spinal cords of SOD1 DJ-1 KO

The nuclear factor erythroid-2 related factor 2 (Nrf2) is a key regulator of the cellular defense systems against oxidative injury. One of the key antioxidant protein that is regulated by Nrf2 is hemoxegenase 1 (HO-1). No significant change in Nrf2 expression was found between WT and DJ-1 KO mice (Fig. 7A). In early disease stages (90 days) Western blot analysis demonstrated increased Nrf2 and HO-1 proteins levels in SOD1 mice compared to WT and DJ-1 KO mice (Fig. 7A, B). In both SOD1 and SOD1 DJ-1 KO mice there is elevation of HO-1 levels, yet in SOD1 DJ-1 KO mice the elevation is more modest (Fig. 7B). As compared to DJ-1 KO mice, the levels of HO-1 in SOD1 DJ-1-KO mice is elevated since as in SOD1 mice, the oxidative and inflammatory insults are greater in ALS mice. Nrf2 is a key regulator of HO-1, yet the transcription of HO-1 is regulated by various oxidative and inflammatory signals (including activator protein-1, and nuclear factor-kappa B, and some of their upstream kinases, mitogen-activated protein kinases, phosphatidylinositol 3-kinase, or protein kinases A, C). Since DJ-1 is needed for activation of the defensive Nrf2 system, lack of DJ-1 in SOD1 DJ-1 KO mice resulted in a blunted response and increased their vulnerability to the disease process and led to augmented clinical deterioration.

Damaged glutamate transport leading to increased glutamate excitotoxicity is a leading hypothesis in the disease mechanisms of ALS. Using real time PCR we measured glutamate transporter-1 expression. We found a significant reduction in glutamate transporter-1 mRNA levels in both SOD1 and SOD1 DJ-1 KO mice as compared to WT or DJ-1 KO mice (to 45±2.6% and 52±5%, respectively). No significant difference was found between SOD1 and SOD1 DJ-1 KO mice.

### DJ-1-derived peptide protects *in vitro* motor neuron-like cells against neurotoxic insults

**Fig 8:**
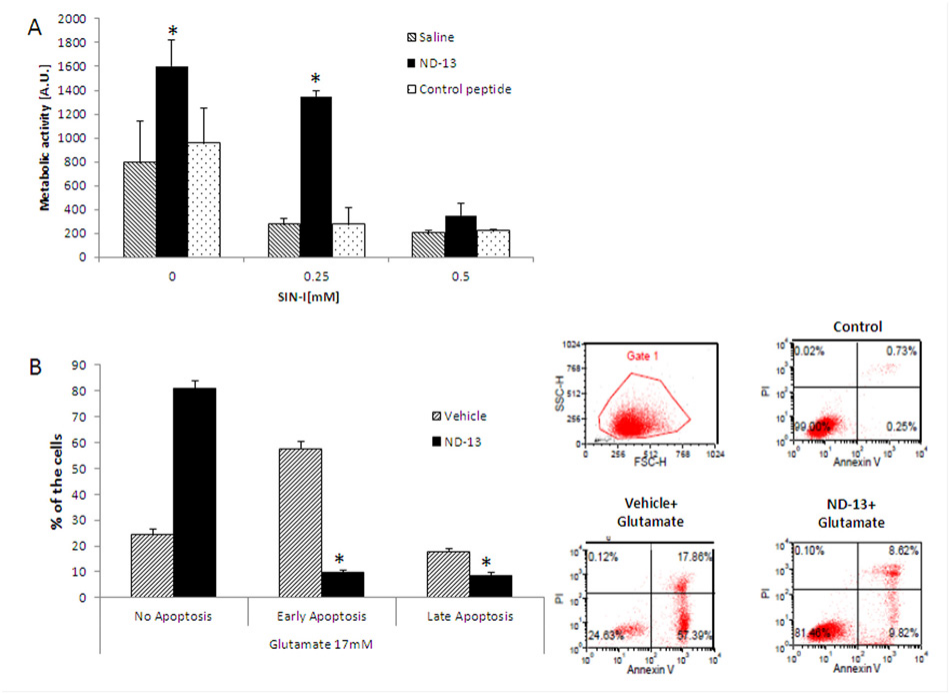
Pre-treatment with DJ-1 derived peptide (ND-13) protected against SIN-1 and glutamate-induced toxicity *in vitro*. NSC-34 cells were exposed to peroxynitrite ion generator 3-morpholinosydnonimine (SIN-1), and cell survival was evaluated by Alamar blue assay (A). The experiment was reapeted 3 times and the results are presented as averages ± SD. * p<0.05. Glutamate-induced apoptosis was measured by Annexin V-FITC, PI staining using FACS analysis (B). Different stages of apoptosis were differentiated as follows: early apoptosis (Annexin V+/PI−); late apoptosis/necrosis cells (Annexin V+/PI+); and viable cells (Annexin V−/PI−). Histograms represent the proportion of apoptotic cells relative to total cells. Plots were calculated from the histogram distributions (avarages ± SD). # p<0.05; ## p<0.01; ***, ### p<0.001; * vs. control; and # vs. glutamate-treated group.

Motor-neuron-like cell-line NSC-34, was used as a cellular *in vitro* model. Exposure to increasing doses of SIN-I (0–0.5 mM) resulted in cell death (Fig. 8A). We evaluated the neuroprotective effect of DJ-1-based peptide (ND-13) against SIN-1-induced neurotoxicity. In these experiments, ND-13 (10 μM) significantly reduced cell mortality induced by serum deprevationconditions and by SIN-1 exposure while the control (scrambled) peptide had no effect on cell toxicity (Fig. 8A).The ability of ND-13 to protect against glutamate-induced apoptosis was evaluated using Annexin V-FITC and PI double-staining, analyzed by FACS. The various stages of apoptosis were differentiated as follows: early apoptosis (Annexin V+/PI−); late apoptosis/necrosis cells (Annexin V+/PI+); and viable cells (Annexin V−/PI−). Exposure to 17 mM glutamate induced early or late apoptosis in 75.4% of the cells. Pretreatment with DJ-1 derived peptide, ND-13, reduced the cells' apoptosis rate to 16.4% (Fig. 7B). After ND-13 treatment, the percentage of cells at the early apoptosis stages was reduced from 57.4% to 9.1%, while at the late apoptosis stage the percentage was reduced from 17.9% to 7.2% (Fig. 8B).

## Discussion

This study demonstrated that loss of DJ-1 accelerates disease, augments disease severity and shortens survival of SOD1 mice, an animal model of ALS. Clinical surveillance and behavioral motor performance analysis revealed that lack of functional DJ-1 leads to earlier disease onset, increased disease progression and shortened the total life expectancy by 20 days. These changes were found both in male and in female mice. *In vitro* experiments showed that increasing DJ-1 activity, by using a DJ-1-based peptide, protected neuronal cells against neurotoxic insults and increased cell survival. These results are in accordance with previous studies that found alterations in DJ-1 levels and function in animal models of ALS [21] and in the CSF of ALS patients [22]. As far as we know, this is the first report showing that loss of functional DJ-1 dramatically augments the disease severity *in vivo*.

There is a large body of literature showing that an abnormal production of ROS, leading to oxidative damage, contributes to the disease process in human ALS and in its experimental models. Previous studies have demonstrated increased oxidative damage to proteins, lipids, and DNA in ALS post mortem tissues [28]. Evidence for oxidative damage has been found in the spinal cord and in the brain of sporadic ALS cases [29, 30] and SOD1 familial ALS patients [31]. Markers for protein and lipid oxidation were detected in motor neurons, reactive astrocytes, and microglia [32]. Cerebrospinal fluid samples from ALS patients demonstrated increased levels of 8-OHdG (indicative of DNA oxidation), 4-hydroxynonenal (indicative of lipid peroxidation), ascorbate free radical, 3-nitrotyrosine levels and nitrated manganese superoxide dismutase [32, 33]. Increased nitration and oxidation have also been demonstrated in animal models of ALS-increased protein oxidation and nitration [34], lipid oxidation [35], and increased oxidative damage to DNA in spinal cord, cortex, and striatum [36]. Protein carbonylation measured immunologically revealed an almost exponential increase in these post translational modifications between 90 and 120 days in the SOD1 mice [34], indicating the increased oxidative damage with disease progression.

DJ-1 plays an important role in the oxidative stress response. *In vitro* and *in vivo* studies suggest that vulnerability to oxidative insults is related to DJ-1 expression levels. DJ-1 knockdown by short-interfering RNA rendered neuroblastoma cells susceptible to hydrogen peroxide-, 1-methyl-4-phenylpyridinium-, rotenone, or 6-hydroxydopamine-induced cell death [37, 38]. Cell death induced by oxidative insults was dramatically reduced by overexpression of wild type DJ-1 [19]. *In vivo* studies in mice and in drosophila confirmed that knock down of DJ-1 increased vulnerability to oxidative insults [39]. These results imply that DJ-1 has a role in the cellular defense mechanism against oxidative stress.

We have previously shown that there are changes in DJ-1 expression levels and oxidized isoforms in brains and spinal cords of SOD1 transgenic mice [21]. Increased DJ-1 mRNA and protein levels appeared early in the pre-symptomatic disease stage [21]. We also detected an increase in DJ-1 acidic isoforms in diseased mice, indicating the presence of oxidized forms of DJ-1 in the CNS of SOD1 mice. The percentage of acidic DJ-1 isoforms of total DJ-1 protein increased with disease progression, implying ongoing oxidative damage. Yamashita et al. [22] also showed that DJ-1 protein levels were upregulated in the motor neurons of the spinal cords of FALS mice throughout the lifespan, as compared to wild-type littermates. Biochemical approaches revealed that mutant SOD1 formed complexes with DJ-1 in the cell lysates. Exogenous DJ-1 resulted in an increase in cell viability and a reduction in cell toxicity in mutant SOD1-transfected neuronal cell lines [22]. CSF analysis for DJ-1 protein found that human patients suffering from sporadic ALS had significantly higher DJ-1 levels in the CSF as compared to control subjects [22]. Recently, Knippenberg et al [40] studied the expression of DJ-1 and PINK-1 in samples from sporadic human ALS patients and from mutant SOD1 transgenic mice and found potential pathophysiologic roles for these proteins in both mutant SOD1 transgenic mice and in sporadic ALS. Collectively, these findings strongly support the mechanistic concept that oxidative injury in the CNS leads to an alteration in DJ-1 levels and in isoforms at early stages of the disease. The current results support the hypothesis that upregulation of DJ-1 in the CNS of young SOD1 mice offers protection aimed to counteract the ongoing detrimental disease process, since a more severe and aggressive disease process was observed in DJ-1 knockout mice. Lack of functional DJ-1, as in the double transgenic DJ-1 KO SOD1 mice, resulted in accelerated damage to the CNS, an accelerated disease course and shortened survival time.

Mutant SOD1 transgenic mice are invaluable for mechanistic study of ALS and development/evaluation of therapeutic targets, yet it is imperative to remember that most ALS patients suffer from sporadic disease and therefore the applicability of knowledge obtained from this model should be carefully evaluated. With this in mind, we were encouraged by data demonstrating oxidative damage in the brains, spinal cords, and CSF of sporadic ALS patients [28–30, 32, 33] and alterations in DJ-1 levels in the CSF of sporadic ALS patients [22].

Presence of functuinal DJ-1 is important for the activation of the Nrf2 system. Nrf2 is a master regulator of redox homeostasis and induces the regulated expression of numerous genes involved in ROS detoxification. These genes encode hemeoxygenase-1 (HO-1), NAD(P)H quinine oxidoreductase 1 (NQO-1), GPx, glutathione reductase (GR), and the catalytic and modulatory subunits of g-glutamyl cysteine ligase (GCL-C and GCL-M, respectively), among many others [41]. Mimoto et al. [42] have recently found an impairment of the Nrf2 system activity in SOD1 mice. They showed that while Nrf2 dramatically increased in the anterior lumbar cord with the accumulation in the motor neurons nucleus, downstream stress response proteins such as HO-1, demonstrated only a small increase. Our results also revealed that loss of DJ-1 function augmented the impairment of Nrf2 system activation and that the double transgenic SOD1 DJ-1 KO mice showed decreased spinal Nrf2 and HO-1 expression levels as compared to SOD1 mice.

The abnormal glutamate metabolism accompanied by the selective loss of the astroglial glutamate transporter-1 GLT-1 (and its human counterpart EAAT2) have been observed in sporadic and familial ALS patients as well as in mutant SOD1 animal models. Our results support the loss of GLT-1 in lumbar spinal cords of both SOD1 and DJ-1 KO SOD1. No significant differences in gene expression were observed between these transgenic mice although*in vitro*, the DJ-1 related peptide showed protective effects against glutamate toxicity. *In vitro*, ND-13, the DJ-1 derived peptide, protected neuronal cell lines against neurotoxic insults implicated in the pathogenesis of ALS, SIN-I and glutamate toxicity. Further studies are needed to verify the ability of this peptide to alter the disease course *in vivo*, and should be examined in different models of the disease since the results presented herein were obtained in mutated SOD1 model of the disease These results imply that DJ-1 is a possible therapeutic target in ALS(at least with mutated SOD1) and encourage further research on the mechanisms involved in the neuroprotective properties of DJ-1 in this devastating disease.
